# Association of balance impairment with risk of incident cardiovascular diseases among older adults

**DOI:** 10.1186/s40001-023-01426-7

**Published:** 2023-10-24

**Authors:** Hye Jun Kim, Seogsong Jeong, Michelle J. Suh, Yun Hwan Oh

**Affiliations:** 1 Department of Biomedical Sciences, Seoul National University College of Medicine, Seoul, 03082 South Korea; 2 Department of Biomedical Informatics, CHA University School of Medicine, CHA University, Seongnam, 13488 South Korea; 3 Department of Otorhinolaryngology, Jeju National University Hospital, Jeju National University College of Medicine, Jeju, 63241 South Korea; 4 Department of Family Medicine, Chung-Ang University Gwangmyeong Hospital, Chung-Ang University College of Medicine, Gwangmyeong, 14353 South Korea

**Keywords:** Postural balance, One-leg standing, Public health, Cardiovascular diseases, Coronary heart disease, Stroke

## Abstract

**Background:**

Rapid decline in balance is a hallmark of aging, elevating the risk of falls and other age-related geriatric illnesses among older adults.

**Objective:**

Our aim was to assess whether impairment in balance function is associated with the risk of incident CVD in older adults.

**Design:**

Retrospective cohort analysis.

**Participants:**

A total of 129,024 participants who had undergone health screening between 2002 and 2009 were derived from the National Health Insurance Service-Senior cohort.

**Main measures:**

Balance impairment was evaluated using the open-eyes one-leg standing (OLS) test. The association between balance impairment and incident CVD was analyzed using the Cox proportional hazards regression model. All participants were followed up with until either the date of the first incident of CVD, death, or 31 December 2019.

**Key results:**

Those with abnormal balance function (< 10 s in OLS test) had a higher risk of CVD (adjusted hazard ratio [aHR] 1.23, CI 1.16–1.31). The association was significant in both the obese and the non-obese, but it seemed to be more pronounced in the latter. Results were supported by sensitivity analyses that did not include cases of CVD development in the first 1, 2, or 3 years and that used a different criterion to define balance dysfunction (< 9 s in OLS test).

**Conclusions:**

Older adults with balance impairment were found to have an increased risk of incident CVD. Patients with impaired balance function may be a high-risk population who require preventive managements against CVD.

**Supplementary Information:**

The online version contains supplementary material available at 10.1186/s40001-023-01426-7.

## Introduction

Despite a gradual decline in cardiovascular diseases (CVD) prevalence in developed countries due to therapeutic advances and preventative efforts, CVD remains a significant burden in an aging population [1]. CVD not only causes progressive dysfunction in older adults, but also remain as the leading cause of death in this age group [2]. Evidence-based clinical practice guidelines are frequently used and generally followed by practitioners in the field of cardiovascular care [3]. Although the identification and implementation of CVD risk factors according to these guidelines has greatly benefited the general population, such recommendations may be less reliable in older adults, as clinical complexities associated with aging are more likely to confound standard precepts [4].

Along with aging, the gradual biologic changes associated with longevity are favorable to the onset of CVD and other age-related geriatric conditions such as frailty, sarcopenia, and falls [2, 5]. For older adults, mobility impairment is associated with a lower quality of life, an increased risk of institutionalization, and premature death [6]. While good mobility necessitates the ability to maintain one's balance while engaging in a variety of activities [7], aging can cause changes that lead to dysfunctional balancing, independently of illness or medication [8, 9]. Maintaining balance requires comprehensive contributions from the vestibular, visual, and proprioceptive systems [10].

Recently, a significant inverse association between balance function and CVD-related mortality risk was reported [7]. Similarly, a link between locomotor dysfunction and an increased risk of CVD has been found in the adults of mean age 66 [11]. Despite ongoing efforts to determine the influence of mobility dysfunction on the CVD risk, the presence of balance dysfunction is likely underestimated in clinical practice, and most investigations are focused on the mortality risk of CVD [6, 12]. Hence, we conducted this study to provide an evidence whether balance function increases the risk of CVD and the extent of risk elevation using the one-leg standing (OLS) test, which is an easy way to self-recognize balance function and CVD risk.

## Methods

### Study population

Data for this retrospective cohort study were drawn from the Korea National Health Insurance Service (NHIS)-Senior Cohort. The elucidations of the cohort are described in detail elsewhere [13]. The NHIS provides compulsory insurance services regarding all aspects of medical healthcare for Korean citizens. Information on lifestyle behaviors, serological characteristics, and anthropometric measurements have been collected by the NHIS alongside demographic information, drug prescription records, treatment notes, and the results of health screening examinations.

**Fig. 1:**
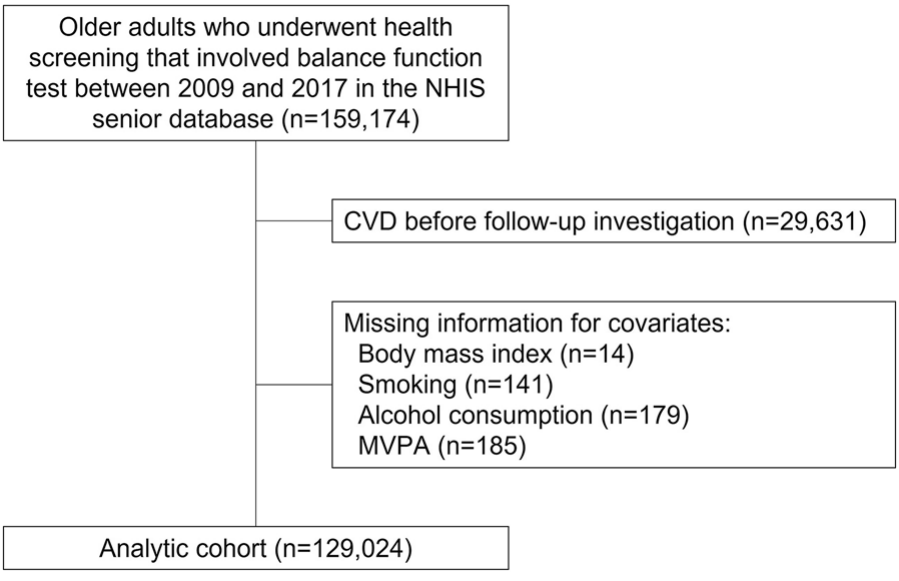
Flow diagram for the inclusion of study participants

We analyzed data from 159,174 people who took a balance function test as part of a health screening between 2009 and 2017. First, we filtered out the people who already had a CVD diagnosis before the follow-up began (*n* = 29,631), as well as those with missing information for covariates (*n* = 519). Finally, a total of 129,024 people aged 66 without a known history of CVD were included in the final analytic cohort (Fig. 1). The study population’s age is limited to 66 years because the NHIS only provides the OLS test for those aged 66. This study was conducted in accordance with STROBE guidelines [14]. This study was approved as exempt from Institutional Review Board of Jeju National University Hospital (approval No. JNUH IRB File No.2021-07-008). Since the database was made anonymous in accordance with strict confidentiality guidelines, it was unnecessary to obtain informed consents.

### Follow-up for CVD

Starting on January 1, 2009, all participants were followed up until either CVD diagnosis, death, or 31 December, 2019. The presence of CVD was determined by whether or not a participant had been hospitalized for two or more days due to coronary heart disease (CHD) (International Classification of Diseases Tenth Revision [ICD-10] codes, I20-I25) or stroke (ICD-10 codes, I60-I69) according to the American Heart Association guidelines [15]. Several prior research have relied on the NHIS's operational definition of CVD, and is considered accurate [16].

### OLS test

The OLS test was applied to evaluate the balance function of the participants. This test is considered highly reliable and predictable for certain outcomes, such as falls and incident disability, and was carried out by medical professionals for those who could be standing without assistance only [17]. The OLS test measured the time an individual could stand on one leg with eyes open. Normal, cautious, and abnormal balance functions were stratified according to ≥ 20 s (s), between 10 to 19 s, and < 10 s from the OLS test.

### Key variables

Potential confounding factors for the adjusted analyses included age (continuous; years), sex (categorical; men and women), insurance premium as a proxy for household income (categorical; upper half and lower half), body mass index (BMI; continuous; kg/m^2^), type 2 diabetes (DM; categorical; yes and no), hypertension (categorical; yes and no), dyslipidemia (categorical; yes and no), smoking (categorical; never, past, and current), alcohol consumption (categorical; yes and no), moderate-to-vigorous physical activity (MVPA; categorical; 0, 1–2, 3–4, and ≥ 5 times/week), and Charlson comorbidity index (CCI; categorical; 0, 1–2, and ≥ 3). MVPA was calculated by moderate-intensity physical activity plus vigorous-intensity physical activity. DM, hypertension, and dyslipidemia were considered present if a participant had the ICD-10 codes (DM, E10 to E14; hypertension, I10; dyslipidemia, E78) records between 2002 and 2008 and prescription records of antidiabetic, antihypertensive, and antidyslipidemic drugs in the same time period. We calculated the CCI as elucidated in a previous study [18].

### Statistical analysis

To assess the association between balance impairment and incident CVD, we used the Cox proportional hazards regression. The adjusted hazard ratio (aHR) and 95% confidence interval (CI) were calculated after adjusting for age and sex solely in the first adjustment model. In addition, we included age, sex, household income, BMI, DM, hypertension, dyslipidemia, alcohol consumption, smoking, MVPA, and CCI as adjustment variables in the final adjustment model.

The incidence of CVD was calculated after dividing events by 1000 person-years (PY). We carried out sensitivity analysis to ensure that the primary results were not influenced by other potential reasons. To support the primary results, on-developing CVD cases at the time of follow-up were excluded by excluding CVD cases that occurred within 1, 2, or 3 years since the first date of follow-up.

Stratified analyses were performed to testify potential interactions among balance function, CVD, and stratification variables. The restricted cubic splines (RCS) were drawn by setting 3 knots at the 5th, 50th, and 95th percentiles to visualize the association between continuous OLS seconds and incident CVD. A two-sided *P* < 0.05 was considered statistically significant. Statistical analyses were performed using R software version 3.3.3 (R foundation for Statistical Computing, Vienna, Austria) and SAS version 9.4 (SAS Institute Inc., Cary, NC, USA).

## Results

### Participant characteristics

Descriptive baseline characteristics of the analytic cohort are presented in Table 1. There were 88,000, 26,186, and 14,838 older adults with normal, cautious, and impaired balance functions. Those with balance dysfunction were more tended to be women sex with lower household income, DM, hypertension, dyslipidemia, comorbidities, and hearing impairment who have higher BMI, higher blood pressures, higher fasting serum glucose, and lower frequency of MVPA.

**Table 1:** Baseline characteristics of the participants who underwent health screening according to the balance function

Characteristic	Normal, ≥ 20 s (*n* = 88,000)	Cautious, 10–19 s (*n* = 26,186)	Abnormal, < 10 s (*n* = 14,838)	*P* value
Age, years	66 (66–66)	66 (66–66)	66 (66–66)	0.282
Sex, *n* (%)				< 0.001
Men	43,663 (49.6)	10,157 (38.8)	4930 (33.2)	
Women	44,337 (50.4)	16,029 (61.2)	9908 (66.8)	
Household income, *n* (%)				< 0.001
Upper half	52,212 (59.3)	15,181 (58.0)	8286 (55.8)	
Lower half	35,788 (40.7)	11,005 (42.0)	6552 (44.2)	
Body mass index, kg/m^2^	23.9 (22.1–25.8)	24.3 (22.5–26.4)	24.7 (22.6–27.0)	< 0.001
Waist circumference, cm	82 (77–88)	83 (78–89)	84 (79–90)	< 0.001
Systolic blood pressure, mmHg	128 (118–136)	130 (119–138)	130 (120–139)	< 0.001
Diastolic blood pressure, mmHg	78 (70–82)	79 (70–83)	79 (70–84)	< 0.001
Fasting serum glucose, mg/dL	98 (90–109)	99 (90–111)	99 (91–114)	< 0.001
Total cholesterol, mg/dL	195 (171–221)	196 (171–223)	195 (169–223)	0.003
Smoking status, *n* (%)				< 0.001
Never	58,952 (67.0)	19,326 (73.8)	11,265 (75.9)	
Previous	17,861 (20.3)	3755 (14.3)	1863 (12.6)	
Current	11,187 (12.7)	3105 (11.9)	1710 (11.5)	
Alcohol consumption, *n* (%)				< 0.001
Yes	27,486 (31.2)	6981 (26.7)	3631 (24.5)	
No	60,514 (68.8)	19,205 (73.3)	11,207 (75.5)	
MVPA, *n* (%)				< 0.001
0 time/week	43,428 (49.4)	14,550 (55.6)	9170 (61.8)	
1–2 time/week	10,376 (11.8)	2806 (10.7)	1508 (10.2)	
3–4 time/week	11,132 (12.7)	2838 (10.8)	1380 (9.3)	
≥ 5 time/week	23,064 (26.2)	5992 (22.9)	2780 (18.7)	
Hypertension, *n* (%)	28,209 (32.1)	9571 (36.6)	5953 (40.1)	< 0.001
Diabetes, *n* (%)	8,662 (9.8)	3222 (12.3)	2316 (15.6)	< 0.001
Dyslipidemia, *n* (%)	13,866 (15.8)	4546 (17.4)	2762 (18.6)	< 0.001
Charlson comorbidity index, *n* (%)				< 0.001
0	15,120 (17.2)	3983 (15.2)	2037 (13.7)	
1	20,326 (23.1)	5569 (21.3)	2954 (19.9)	
≥ 2	52,554 (59.7)	16,634 (63.5)	9847 (66.4)	
Hearing impairment, *n* (%)				< 0.001
Yes	994 (1.1)	369 (1.4)	276 (1.9)	
No	87,006 (98.9)	25,817 (98.6)	14,562 (98.1)	

Data are mean (standard deviation) unless indicated otherwise

### Association between balance impairment and risk of CVD

**Fig. 2:**
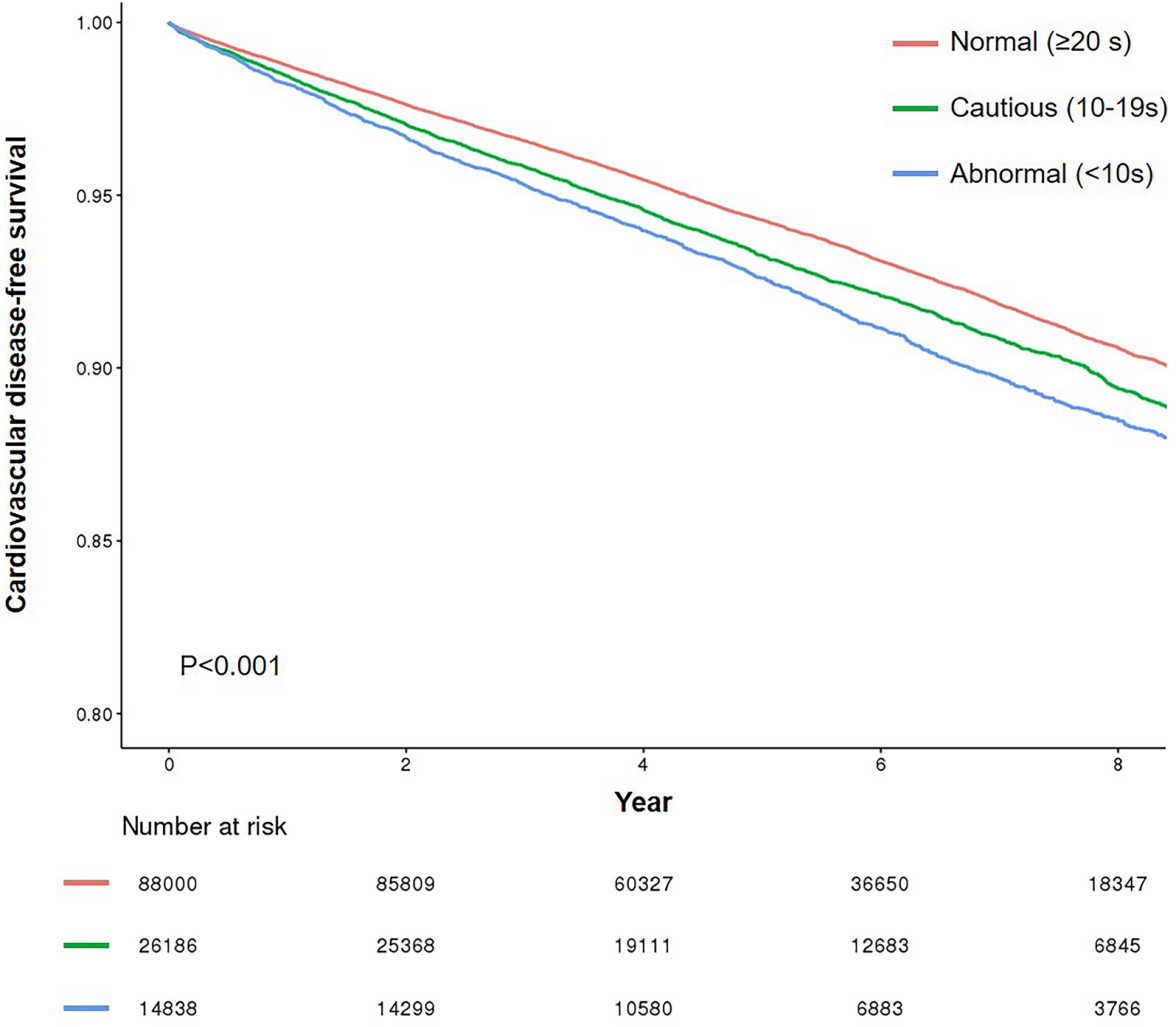
Kaplan–Meier estimation of cardiovascular disease risk according to the balance function

**Fig. 3:**
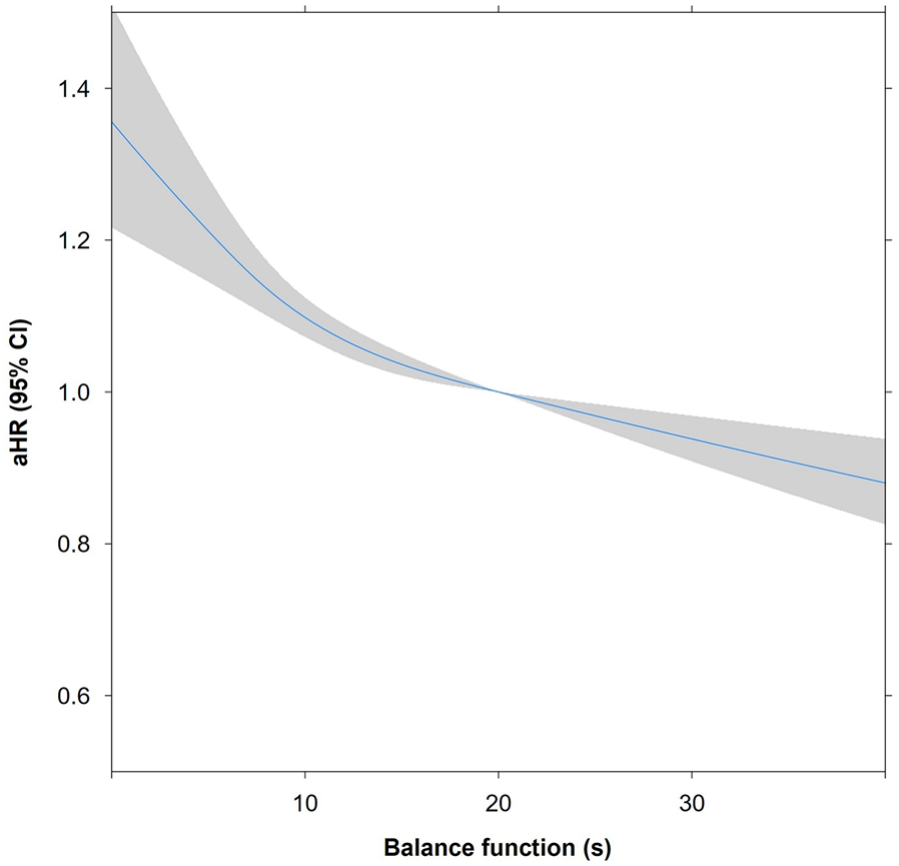
Restricted cubic spline used for evaluating the association between balance function and risk of incident cardiovascular disease among older adults. Adjusted hazard ratio calculated after adjustments for age, sex, household income, body mass index, hypertension, diabetes, dyslipidemia, smoking, alcohol consumption, moderate-to-vigorous physical activity, and Charlson comorbidity index

A total of 9587 cases of new onset CVD were identified during the 737,500 person-years of follow-up. There was a strong association between lower balance function and an increased risk of CVD (Table 2; *P* for trend < 0.001). Compared with participants with normal balance function, those with impaired balance function had an elevated risk of CVD (aHR, 1.23; 95% CI 1.16–1.31). Both obese (aHR, 1.17; 95% CI 1.07–1.28) and non-obese groups (aHR, 1.30; 95% CI 1.20–1.41) showed similar results. In addition, the associations of balance impairment with CHD (aHR, 1.23; 95% CI 1.16–1.31) and stroke (aHR, 1.33; 95% CI 1.23–1.43) were significant (Table 3). Also, in line with the main findings of this study, the Kaplan–Meier cumulative risks for overall CVD were higher in the abnormal balance group than the normal balance function group (Fig. 2). Additionally, we identified the risk-increasing effect on CVD by shorter OLS in a continuous manner (Fig. 3).

**Table 2:** Association of balance function with incident cardiovascular disease

	Normal (≥ 20 s)	Cautious (10–19 s)	Abnormal (< 10 s)	*P* for trend
Overall participant, *n*	88,000	26,186	14,838	
Event (%)	6,054 (6.9)	2,190 (8.4)	1,343 (9.1)	
Person-year	493,593	156,745	87,162	
Incidence/1000 person-year	12.3	14.0	15.4	
HR (95% CI)	1.00 (Reference)	1.13 (1.08–1.19)	1.25 (1.18–1.33)	< 0.001
aHR (95% CI)^a^	1.00 (Reference)	1.19 (1.13–1.25)	1.34 (1.27–1.43)	< 0.001
aHR (95% CI)^b^	1.00 (Reference)	1.14 (1.08–1.19)	1.23 (1.16–1.31)	< 0.001
Obese participant, *n*	31,538	10,971	7,010	
Event (%)	2,291 (7.3)	954 (8.7)	608 (8.7)	
Person-year	174,824	64,792	40,868	
Incidence/1000 person-year	13.1	14.7	14.9	
HR (95% CI)	1.00 (Reference)	1.12 (1.04–1.21)	1.13 (1.04–1.24)	0.002
aHR (95% CI)^a^	1.00 (Reference)	1.18 (1.10–1.28)	1.24 (1.13–1.36)	< 0.001
aHR (95% CI)^b^	1.00 (Reference)	1.15 (1.07–1.24)	1.17 (1.07–1.28)	< 0.001
Non-obese participant, *n*	56,462	15,215	7,828	
Event (%)	3,763 (6.7)	1,236 (8.1)	735 (9.4)	
Person-year	318,769	91,952	46,294	
Incidence/1000 person-year	11.8	13.4	15.9	
HR (95% CI)	1.00 (Reference)	1.13 (1.06–1.21)	1.34 (1.24–1.45)	< 0.001
(95% CI)^a^	1.00 (Reference)	1.17 (1.10–1.25)	1.41 (1.30–1.53)	< 0.001
aHR (95% CI)^b^	1.00 (Reference)	1.13 (1.06–1.20)	1.30 (1.20–1.41)	< 0.001

HR calculated using Cox proportional hazards regression

Acronym: *HR* hazard ratio, *CI* confidence interval, *aHR* adjusted hazard ratio

^a^Adjusted for age and sex

^b^Adjusted for age, sex, household income, hypertension, diabetes, dyslipidemia, smoking, alcohol consumption, moderate-to-vigorous physical activity, and Charlson comorbidity index

**Table 3:** Association of balance function with incident coronary heart disease and stroke

	Normal (≥ 20 s)	Cautious (10–19 s)	Abnormal (< 10 s)	*P* for trend
Coronary heart disease				
HR (95% CI)	1.00 (Reference)	1.13 (1.08–1.19)	1.25 (1.18–1.33)	< 0.001
aHR (95% CI)^a^	1.00 (Reference)	1.19 (1.13–1.25)	1.34 (1.27–1.43)	< 0.001
aHR (95% CI)^b^	1.00 (Reference)	1.14 (1.08–1.19)	1.23 (1.16–1.31)	< 0.001
Stroke				
HR (95% CI)	1.00 (Reference)	1.17 (1.10–1.24)	1.36 (1.26–1.46)	< 0.001
aHR (95% CI)^a^	1.00 (Reference)	1.20 (1.13–1.28)	1.42 (1.32–1.53)	< 0.001
aHR (95% CI)^b^	1.00 (Reference)	1.16 (1.09–1.24)	1.33 (1.23–1.43)	< 0.001

HR calculated using Cox proportional hazards regression

Acronym: *HR* hazard ratio, *CI* confidence interval, *aHR* adjusted hazard ratio

^a^Adjusted for age and sex

^b^Adjusted for age, sex, household income, hypertension, diabetes, dyslipidemia, smoking, alcohol consumption, moderate-to-vigorous physical activity, and Charlson comorbidity index

Primary results were validated through sensitivity analyses that were performed after excluding CVD events within 1, 2, and 3 years since the beginning of the follow-up (Additional file 1: Tables S1, S2).

Subgroup analyses on the association of balance functions with risk of incident CVD are shown in Additional file 1: Table S3. Older adults were stratified according to sex, household income, DM, hypertension, dyslipidemia, MVPA, and hearing impairment. A higher risk of CVD was observed in the impaired balance function groups except for those with hearing impairment, and we found no significant interactions.

## Discussion

The association between OLS test and the risk of CVD was noted in this study. Participants with impaired balance function had a 1.23-fold increased CVD risk compared to participants with normal balance function after adjusting for potential confounders. Despite older adults with impaired balance function were more likely to develop both CHD and stroke, the balance function-dependent risk was stronger for stroke. These findings may pave a way for further investigation into the relationship between balance function and the risk of CVD, providing supports for effective healthcare initiatives.

In the present study, the proportions of cautious and abnormal groups were 20.3% and 11.5%, respectively, which are lower than a Swedish study of 351 women aged 69–79 years (median, 72.4 years) showed that the median OLS time was 19 s (interquartile range, 1.5–30) and 22.5 s (interquartile range, 2–30) for right-leg and left-leg standing participants with high exercise, respectively [19]. Impaired balance contributes to a wide range of negative health outcomes [20]. However, there is limited evidence regarding balance function and the onset of CVD. A recent study involving 5816 US adults aged over 40 found that those with a balance dysfunction had a 65% higher CVD mortality compared to those with normal balance function, as measured by the modified Romberg standing balance test [7]. In a Japanese cohort study with 1,085 old participants aged 65–89 years who received the OLS test, balance impairment was associated with a 91% increased risk of CVD-related death [21]. Another recent Turkish study involving 44 older adults with a mean age of 59 found that balance function was reduced in patients with chronic heart failure as measured by the Activity-Specific Balance Confidence Scale and the Mini-Balance Evaluation Systems Test [20]. The present study expands current knowledge on association of balance function with incident CVD that cautious or abnormal balance function is associated with a higher risk of CVD among older adults.

A behavior-related mechanism may explain the association between balance function and incident CVD. The prevalence of vestibular dysfunction rise sharply with aging, and this can affect the balance function of individuals [22]. People who experienced symptoms such as dizziness as a result of vestibular impairment were 12 times more likely to fall than those with normal vestibular function [22]. Falls, primarily caused by poor balance can result in long-term immobility and a sedentary lifestyle [23]. Certain sedentary behaviors have been associated with an increased risk of CVD [24, 25]. Furthermore, fear of falling causes older adults to be less active and spend more time sitting than they should, increasing their CVD risk [24]. In a biological manner, the activity of the lipoprotein lipase in the postural muscles of rats has been shown to decrease when they are forced to remain immobile [26]. Low levels of lipoprotein lipase are associated with reduced triacylglycerol uptake, lower plasma HDL cholesterol levels, and higher risk of CVD [26]. In addition, lipid abnormalities are related to an increased risk of atherosclerosis or myocardial infarction in patients with vertigo [27].

Another possible explanation is that frailty caused by balance impairment increases the risk of CVD. Despite the fact that a variety of definitions for quantifying frailty status have been proposed [21], poor physical performance is widely recognized as a hallmark of frailty. Perhaps the increased risk of CVD in frail old people is due to their susceptibility to psychological and physiological stresses [21]. Although there is limited evidence to support frailty to a risk factor for CVD incidence, it has been associated to both clinical and underlying CVD [28]. To better inform clinical practice, further investigation to clarify pathways connecting balance disorder and CVD risk is warranted.

To date, assessing the balance of older adults has not been standard practice in clinical examinations. This could be attributed to a lack of consensus on how to measure balance, as well as a lack of information linking balance test results to clinical outcomes other than falls [6]. Therefore, the findings of this study may be useful for screening individuals at risk of CVD and suggesting prevention and intervention initiatives to lower the risk of CVD [29]. However, the following limitations should be considered when interpreting this study results. First, this study is not exempt to the issue of reverse causality because of its retrospective nature. Future prospective studies would be warranted to determine the causal relationship between balance impairment and the risk of CVD. Second, the OLS test was the only parameter used to assess balance performance in this study. Although OLS has been one of the standard methods for evaluating postural stability for several decades, due to its ease of use and short test time [30, 31], additional studies based on other metrics for measuring balance function may be required to generalize our findings. Third, not all potential confounding factors, such as muscle wasting, physical fitness, and cognitive impairment could be considered for the adjusted analyses due to the unavailability of information. Lastly, our study determined the cause-specific aHR for the risk of CVD according to the OLS test results. Evaluating subdistribution hazard ratio that set non-CVD death as a competing event may be necessary to better determine the association of balance function with the risk of CVD.

Balance impairment was associated with an increased risk of incident CVD in this nationally representative cohort study of Korean older adults. As the prevalence of balance impairments rises with aging, assessing balance function in clinical and research settings may be considered to better estimate the risk of CVD. Such assessment may allow better referrals to specialists for individual-level healthcare plans.

### Supplementary Information

**Additional file 1: Table S1.** Sensitivity analyses on the association of balance function with incident cardiovascular disease. **Table S2.** Sensitivity analysis on association of balance function test with incident cardiovascular disease. **Table S3.** Stratified analyses on the association of balance function test with incident cardiovascular disease.

## Data Availability

The datasets generated during and/or analyzed during the current study are available from the corresponding author on reasonable request.

## References

[1] Virani SS, Alonso A, Aparicio HJ (2021). Heart disease and stroke statistics-2021 update: a report from the American heart association. Circulation.

[2] O'Neill DE, Forman DE (2021). Cardiovascular care of older adults. BMJ.

[3] Jeong SM, Choi S, Kim K (2018). Effect of change in total cholesterol levels on cardiovascular disease among young adults. J Am Heart Assoc.

[4] Benjamin EJ, Muntner P, Alonso A (2019). Heart disease and stroke statistics-2019 update: a report from the American heart association. Circulation.

[5] Wright RM, Sloane R, Pieper CF (2009). Underuse of indicated medications among physically frail older US veterans at the time of hospital discharge: results of a cross-sectional analysis of data from the geriatric evaluation and management drug study. Am J Geriatr Pharmacother.

[6] Araujo CG, de Souza E, Silva CG, Laukkanen JA (2022). Successful 10-second one-legged stance performance predicts survival in middle-aged and older individuals. Br J Sports Med.

[7] Cao C, Cade WT, Li S, McMillan J, Friedenreich C, Yang L (2021). Association of balance function with all-cause and cause-specific mortality among US adults. JAMA Otolaryngol Head Neck Surg.

[8] Dutil M, Handrigan GA, Corbeil P (2013). The impact of obesity on balance control in community-dwelling older women. Age.

[9] Bergen G, Stevens MR, Burns ER (2016). Falls and fall injuries among adults aged ≥65 years—United States, 2014. MMWR Morb Mortal Wkly Rep.

[10] Kahiel Z, Grant A, Aubin MJ, Buhrmann R, Kergoat MJ, Freeman EE (2021). Vision, eye disease, and the onset of balance problems: the Canadian longitudinal study on aging. Am J Ophthalmol.

[11] Otani K, Takegami M, Fukumori N (2012). Locomotor dysfunction and risk of cardiovascular disease, quality of life, and medical costs: design of the locomotive syndrome and health outcome in Aizu cohort study (LOHAS) and baseline characteristics of the study population. J Orthop Sci.

[12] Denfeld QE, Turrise S, MacLaughlin EJ (2022). Preventing and managing falls in adults with cardiovascular disease: a scientific statement from the American heart association. Circ Cardiovasc Qual Outcomes.

[13] Kim YI, Kim YY, Yoon JL (2019). Cohort profile: national health insurance service-senior (NHIS-senior) cohort in Korea. BMJ Open.

[14] von Elm E, Altman DG, Egger M (2007). The strengthening the reporting of observational studies in epidemiology (STROBE) statement: guidelines for reporting observational studies. Ann Intern Med.

[15] Mozaffarian D, Benjamin EJ, Writing Group Members (2016). Heart disease and stroke statistics-2016 update: a report from the American heart association. Circulation.

[16] Son JS, Choi S, Kim K (2018). Association of blood pressure classification in Korean young adults according to the 2017 American college of cardiology/American heart association guidelines with subsequent cardiovascular disease events. JAMA.

[17] Vafaei A, Aubin MJ, Buhrmann R, Kergoat MJ, Aljied R, Freeman EE (2018). Interaction between visual acuity and peripheral vascular disease with balance. J Am Geriatr Soc.

[18] Sundararajan V, Henderson T, Perry C, Muggivan A, Quan H, Ghali WA (2004). New ICD-10 version of the Charlson comorbidity index predicted in-hospital mortality. J Clin Epidemiol.

[19] Papp ME, Grahn-Kronhed AC, Rauch Lundin H, Salminen H (2022). Changes in physical activity levels and relationship to balance performance, gait speed, and self-rated health in older Swedish women: a longitudinal study. Aging Clin Exp Res.

[20] Ozcan EB, Saglam M, Vardar-Yagli N (2022). Impaired balance and gait characteristics in patients with chronic heart failure. Heart Lung Circ.

[21] Nofuji Y, Shinkai S, Taniguchi Y (2016). Associations of walking speed, grip strength, and standing balance with total and cause-specific mortality in a general population of Japanese elders. J Am Med Dir Assoc.

[22] Agrawal Y, Carey JP, Della Santina CC, Schubert MC, Minor LB (2009). Disorders of balance and vestibular function in US adults: data from the national health and nutrition examination survey, 2001–2004. Arch Intern Med.

[23] Bruce DG, Devine A, Prince RL (2002). Recreational physical activity levels in healthy older women: the importance of fear of falling. J Am Geriatr Soc.

[24] Yang L, Cao C, Kantor ED (2019). Trends in sedentary behavior among the US population, 2001–2016. JAMA.

[25] Wilmot EG, Edwardson CL, Achana FA (2012). Sedentary time in adults and the association with diabetes, cardiovascular disease and death: systematic review and meta-analysis. Diabetologia.

[26] Hamilton MT, Healy GN, Dunstan DW, Zderic TW, Owen N (2008). Too little exercise and too much sitting: inactivity physiology and the need for new recommendations on sedentary behavior. Curr Cardiovasc Risk Rep.

[27] Santos MD, Bittar RS (2012). Vertigo and metabolic disorders. Int Tinnitus J.

[28] Newman AB, Gottdiener JS, Mcburnie MA (2001). Associations of subclinical cardiovascular disease with frailty. J Gerontol A Biol Sci Med Sci.

[29] D'Silva LJ, Lin J, Staecker H, Whitney SL, Kluding PM (2016). Impact of diabetic complications on balance and falls: contribution of the vestibular system. Phys Ther.

[30] Springer BA, Marin R, Cyhan T, Roberts H, Gill NW (2007). Normative values for the unipedal stance test with eyes open and closed. J Geriatr Phys Ther.

[31] Narozny W, Tretiakow D, Skorek A (2021). Investigations of malfunctions of the vestibular system. JAMA Otolaryngol Head Neck Surg.

