# Supplementary material for: Association of balance impairment with risk of incident cardiovascular diseases among older adults
Source: Eur J Med Res. 2023 Oct 24;28:455. doi: 10.1186/s40001-023-01426-7 (PMC10594686; doi:10.1186/s40001-023-01426-7)
Supplement: Supplementary file 1 — Additional file 1: Table S1. Sensitivity analyses on the association of balance function with incident cardiovascular disease. Table S2. Sensitivity analysis on association of balance function test with incident cardiovascular disease. Table S3. Stratified analyses on the association of balance function test with incident cardiovascular disease. [file 40001_2023_1426_MOESM1_ESM.docx]

**Table S1. Sensitivity analyses on the association of balance function with incident cardiovascular disease**

|  | Normal (≥20 s) | Cautious (10-19 s) | Abnormal (<10 s) | *P* for trend |
| --- | --- | --- | --- | --- |
| 1-year washed out | 1.00 (Reference) | 1.11 (1.05-1.18) | 1.19 (1.12-1.28) | <0.001 |
| 2-year washed out | 1.00 (Reference) | 1.08 (1.02-1.15) | 1.16 (1.08-1.25) | <0.001 |
| 3-year washed out | 1.00 (Reference) | 1.06 (0.99-1.14) | 1.12 (1.03-1.23) | 0.016 |

Data are adjusted hazard ratio calculated using Cox proportional hazards regression after adjustments for age, sex, household income, body mass index, hypertension, diabetes, dyslipidemia, smoking, alcohol consumption, moderate-to-vigorous physical activity, and Charlson comorbidity index.

**Table S2. Sensitivity analysis on association of balance function test with incident cardiovascular disease**

|  | OLS>9 s | OLS≤9 s | *P* value |
| --- | --- | --- | --- |
| Overall participant, n | 114,186 | 14,838 |  |
| Event (%) | 8,244 (7.2) | 1,343 (9.1) |  |
| Person-year | 650,338 | 87,162 |  |
| Incidence/1,000 person-year | 12.7 | 15.4 |  |
| HR (95% CI) | 1.00 (Reference) | 1.21 (1.14-1.28) | <0.001 |
| aHR (95% CI)^a^ | 1.00 (Reference) | 1.29 (1.21-1.36) | <0.001 |
| aHR (95% CI)^b^ | 1.00 (Reference) | 1.19 (1.12-1.26) | <0.001 |

HR calculated using Cox proportional hazards regression.

^a^Adjusted for age and sex.

^b^Adjusted for age, sex, household income, hypertension, diabetes, dyslipidemia, smoking, alcohol consumption, moderate-to-vigorous physical activity, and Charlson comorbidity index.

Acronym: OLS, one-leg standing; HR, hazard ratio; CI, confidence interval; aHR, adjusted hazard ratio.

**Table S3. Stratified analyses on the association of balance function test with incident cardiovascular disease**

|  | Normal (≥20 s) | Cautious (10-19 s) | Abnormal (<10 s) | *P* for trend | *P* for interaction |
| --- | --- | --- | --- | --- | --- |
| Sex |  |  |  |  | 0.152 |
| Men | 1.00 (Reference) | 1.10 (1.03-1.18) | 1.29 (1.18-1.41) | <0.001 |  |
| Women | 1.00 (Reference) | 1.17 (1.09-1.26) | 1.20 (1.10-1.30) | <0.001 |  |
| Household income |  |  |  |  | 0.705 |
| Upper half | 1.00 (Reference) | 1.16 (1.08-1.23) | 1.23 (1.13-1.33) | <0.001 |  |
| Lower half | 1.00 (Reference) | 1.11 (1.03-1.20) | 1.24 (1.13-1.35) | <0.001 |  |
| Hypertension |  |  |  |  | 0.887 |
| Yes | 1.00 (Reference) | 1.14 (1.06-1.23) | 1.26 (1.15-1.37) | <0.001 |  |
| No | 1.00 (Reference) | 1.13 (1.06-1.21) | 1.21 (1.11-1.32) | <0.001 |  |
| Diabetes |  |  |  |  | 0.331 |
| Yes | 1.00 (Reference) | 1.16 (1.03-1.31) | 1.36 (1.20-1.55) | <0.001 |  |
| No | 1.00 (Reference) | 1.13 (1.07-1.20) | 1.20 (1.12-1.28) | <0.001 |  |
| Dyslipidemia |  |  |  |  | 0.444 |
| Yes | 1.00 (Reference) | 1.16 (1.03-1.30) | 1.16 (1.01-1.33) | 0.014 |  |
| No | 1.00 (Reference) | 1.13 (1.07-1.20) | 1.25 (1.17-1.34) | <0.001 |  |
| MVPA, time/week |  |  |  |  | 0.243 |
| 0 | 1.00 (Reference) | 1.15 (1.07-1.23) | 1.18 (1.09-1.28) | <0.001 |  |
| 1-2 | 1.00 (Reference) | 1.09 (0.93-1.26) | 1.18 (0.97-1.43) | 0.187 |  |
| 3-4 | 1.00 (Reference) | 1.17 (1.01-1.35) | 1.32 (1.09-1.59) | 0.006 |  |
| ≥5 | 1.00 (Reference) | 1.11 (1.00-1.24) | 1.41 (1.24-1.62) | <0.001 |  |
| Hearing impairment |  |  |  |  | 0.456 |
| Yes | 1.00 (Reference) | 1.45 (1.02-2.07) | 1.35 (0.90-2.03) | 0.085 |  |
| No | 1.00 (Reference) | 1.13 (1.08-1.19) | 1.23 (1.16-1.31) | <0.001 |  |

Data are adjusted hazard ratio calculated using Cox proportional hazards regression after adjustments for age, sex, household income, body mass index, hypertension, diabetes, dyslipidemia, smoking, alcohol consumption, moderate-to-vigorous physical activity, and Charlson comorbidity index.
